# Weight is More Informative than Body Mass Index for Predicting Postmenopausal Breast Cancer Risk: Prospective Family Study Cohort (ProF-SC)

**DOI:** 10.1158/1940-6207.CAPR-21-0164

**Published:** 2022-03-04

**Authors:** Zhoufeng Ye, Shuai Li, Gillian S. Dite, Tuong L. Nguyen, Robert J. MacInnis, Irene L. Andrulis, Saundra S. Buys, Mary B. Daly, Esther M. John, Allison W. Kurian, Jeanine M. Genkinger, Wendy K. Chung, Kelly-Anne Phillips, Heather Thorne, Heather Thorne, for kConFab, Ingrid M. Winship, Roger L. Milne, Pierre-Antoine Dugué, Melissa C. Southey, Graham G. Giles, Mary Beth Terry, John L. Hopper

**Affiliations:** 1 Centre for Epidemiology and Biostatistics, University of Melbourne, Parkville, Victoria, Australia.; 2 Centre for Cancer Genetic Epidemiology, Department of Public Health and Primary Care, University of Cambridge, Cambridge, United Kingdom.; 3 Precision Medicine, School of Clinical Sciences at Monash Health, Monash University, Clayton, Victoria, Australia.; 4 Genetic Technologies Limited, Fitzroy, Victoria, Australia.; 5 Cancer Epidemiology Division, Cancer Council Victoria, Melbourne, Victoria, Australia.; 6 Departments of Molecular Genetics and Laboratory Medicine and Pathobiology, University of Toronto, Toronto, Ontario, Canada.; 7 Lunenfeld-Tanenbaum Research Institute, Sinai Health System, Toronto, Ontario, Canada.; 8 Department of Medicine and Huntsman Cancer Institute, University of Utah Health Sciences Center, Salt Lake City, Utah.; 9 Department of Clinical Genetics, Fox Chase Cancer Center, Philadelphia, Pennsylvania.; 10 Department of Epidemiology and Population Health, Stanford University School of Medicine, Stanford, California.; 11 Department of Medicine (Oncology), Stanford University School of Medicine, Stanford, California.; 12 Department of Epidemiology, Mailman School of Public Health, Columbia University, New York City, New York.; 13 Herbert Irving Comprehensive Cancer Center, Columbia University Medical Center, New York City, New York.; 14 Departments of Pediatrics and Medicine, Columbia University, New York City, New York.; 15 Department of Medical Oncology, Peter MacCallum Cancer Centre, Melbourne, Victoria, Australia.; 16 Sir Peter MacCallum Department of Oncology, The University of Melbourne, Melbourne, Victoria, Australia.; 17 Research Department, Peter MacCallum Cancer Centre, Melbourne, Victoria, Australia.; 18 Department of Medicine, The University of Melbourne, Melbourne, Victoria, Australia.; 19 Department of Clinical Pathology, The Melbourne Medical School, The University of Melbourne, Melbourne, Australia.

## Abstract

We considered whether weight is more informative than body mass index (BMI) = weight/height^2^ when predicting breast cancer risk for postmenopausal women, and if the weight association differs by underlying familial risk. We studied 6,761 women postmenopausal at baseline with a wide range of familial risk from 2,364 families in the Prospective Family Study Cohort. Participants were followed for on average 11.45 years and there were 416 incident breast cancers. We used Cox regression to estimate risk associations with log-transformed weight and BMI after adjusting for underlying familial risk. We compared model fits using the Akaike information criterion (AIC) and nested models using the likelihood ratio test. The AIC for the weight-only model was 6.22 units lower than for the BMI-only model, and the log risk gradient was 23% greater. Adding BMI or height to weight did not improve fit (ΔAIC = 0.90 and 0.83, respectively; both *P* = 0.3). Conversely, adding weight to BMI or height gave better fits (ΔAIC = 5.32 and 11.64; *P* = 0.007 and 0.0002, respectively). Adding height improved only the BMI model (ΔAIC = 5.47; *P* = 0.006). There was no evidence that the BMI or weight associations differed by underlying familial risk (*P >* 0.2). Weight is more informative than BMI for predicting breast cancer risk, consistent with nonadipose as well as adipose tissue being etiologically relevant. The independent but multiplicative associations of weight and familial risk suggest that, in terms of absolute breast cancer risk, the association with weight is more important the greater a woman's underlying familial risk.

**Prevention Relevance:**

Our results suggest that the relationship between BMI and breast cancer could be due to a relationship between weight and breast cancer, downgraded by inappropriately adjusting for height; potential importance of anthropometric measures other than total body fat; breast cancer risk associations with BMI and weight are across a continuum.

## Introduction

The relationship between body composition and breast cancer risk has been the subject of considerable interest, and obesity is considered an established breast cancer risk factor for postmenopausal women (1). Many studies have considered body mass index (BMI) = weight/(height)^2^ alone, not weight and height as separate entities. Considering BMI alone assumes that height, a risk factor on its own (2, 3), has instead a protective association with breast cancer after adjusting for weight, and of a specific magnitude (3).

In interpreting findings and considering their implications for prevention, it is generally assumed that the breast cancer risk association with BMI is a reflection of total body fat (1) and in particular obesity (4). The correlation between BMI and percentage body fat (measured by Dual-energy X-ray absorptiometry) is around 0.8, decreasing with age (5). However, considering BMI alone could be a self-fulfilling prophecy, seeking evidence consistent with a predetermined position.

It is plausible that other anthropometric aspects could also be important for breast cancer risk. For example, MacInnis and colleagues found that breast cancer risk is predicted, at least 15 or more years postmenopause, by multiple measures of body size (including waist circumference, hip circumference, fat mass, and fat-free mass; ref. 6). Because BMI does not necessarily reflect only total body fat, it is an open question as to whether weight and even other body measures might be better predictors of postmenopausal breast cancer risk than BMI alone.

We previously conducted a prospective study of breast cancer risk using a cohort with a wide range of ages and familial risk and found that the BMI association depended on menopausal status. From fitting the BMI association with breast cancer as a function of both age and menopausal status we observed a nonsignificant negative association for premenopausal women and a positive association for postmenopausal women. After adjusting for the interaction between menopausal status and BMI, there was no association with age (7). We also found that there was no interaction, on the multiplicative scale, between BMI and a continuous measure of underlying familial risk, consistent with the multiplicative BMI risk association not differing by familial risk. We then explained that, as a consequence, on the absolute scale of breast cancer risk the greater a woman's underlying familial risk the more important must be her BMI.

In this paper, we have focused on postmenopausal women and asked if our conclusions about BMI also applied to weight, with or without adjusting for height. We also considered whether the weight association was independent of a woman's underlying familial risk, as we had previously found for the BMI association.

## Materials and Methods

### Subjects

We used data from the breast cancer Prospective Family Study Cohort (ProF-SC), which pooled data from the Breast Cancer Family Registry (BCFR) Cohort and the Kathleen Cuningham Foundation Consortium for research into Familial Breast Cancer (kConFab) Follow-up Study (8). The BCFR is a collaboration of six breast cancer family studies from the USA, Canada, and Australia, and kConFab is an Australian and New Zealand breast cancer family study. Both family cohorts used the same baseline questionnaire for affected and unaffected relatives and conducted regular follow-ups. Extensive details on the sample and methodology have been published (7).

In this study, we included adult women who were postmenopausal and under the age of 79 years at baseline, had been breast cancer–free for at least 3 months after completion of the baseline questionnaire, and had not had a bilateral risk-reducing mastectomy. All the risk-predicting information used was collected at baseline. Cancer diagnoses were updated at regular follow-ups and cancer registry linkages (81% of incident breast cancer were confirmed from pathology reports). The underlying familial risk was based on the predicted 1-year risk of breast cancer based on pedigree information and *BRCA1* and *BRCA2* mutation status information, wherever available, using the Breast and Ovarian Analysis of Disease Incidence and Carrier Estimation Algorithm (BOADICEA) version 3 (9).

A total of 6,761 women from 2,364 families with complete self-reported data on height and weight were included. The mean age at baseline was 61.22 years (SD = 9.41) and the mean duration of follow-up was 11.45 years (SD = 5.39). During the follow-up, there were 416 incident breast cancers diagnosed (mean age at diagnosis 71.25 years, SD = 9.91). Details of participants are shown in Table 1.

**Table 1.** Baseline characteristics of postmenopausal women with unadjusted HR and 95% CI for breast cancer risk.

	Unaffected		Affected				
	*N* = 6,345		*N* = 416				
Variables	Number	%	Number	%	HR	95% CI	*P*
Age at baseline (years)							
18–49	509	8.02	37	8.89	1	(Referent)	
50–59	2,202	34.70	164	39.42	0.67	0.38–1.17	0.156
60–69	2,171	34.22	166	39.90	0.60	0.32–1.12	0.109
70–79	1,333	21.01	36	8.65	0.34	0.15–0.77	0.010
1-year BOADICEA risk (%)							
Q1: 0.00–0.38	1,593	25.11	81	19.47		(Referent)	
Q2: 0.39–0.50	1,622	25.56	94	22.60	1.15	0.84–1.56	0.383
Q3: 0.51–0.66	1,530	24.11	82	19.71	1.11	0.80–1.56	0.535
Q4: 0.67–7.94	1,564	24.65	156	37.50	2.06	1.53–2.78	<0.001
Missing	36	0.57	3	0.72			
Weight (kg)							
Q1: 37–60	1,748	27.55	87	20.91		(Referent)	
Q2: 61–68	1,539	24.26	93	22.36	1.14	0.85–1.52	0.388
Q3: 69–78	1,475	23.25	111	26.68	1.42	1.07–1.88	0.014
Q4: 79–163	1,583	24.95	125	30.05	1.46	1.11–1.92	0.007
Height (m)							
Q1: 1.14–1.57	2,059	32.45	102	24.52	1	(Referent)	
Q2: 1.58–1.63	1,870	29.47	138	33.17	1.47	1.13–1.91	0.004
Q3: 1.64–1.68	1,469	23.15	93	22.36	1.23	0.93–1.62	0.153
Q4: 1.69–2.03	947	14.93	83	19.95	1.61	1.20–2.16	0.001
BMI (kg/m^2^)							
Q1: 14.69–22.86	1,586	25.00	86	20.67		(Referent)	
Q2: 22.86–25.91	1,590	25.06	102	24.52	1.11	0.83–1.49	0.466
Q3: 25.95–29.69	1,599	25.20	104	25.00	1.15	0.86–1.52	0.349
Q4: 29.71–58.86	1,570	24.74	124	29.81	1.38	1.04–1.83	0.024
History of benign breast disease							
No	4,297	67.72	235	56.49	1	(Referent)	
Yes	1,906	30.04	171	41.11	1.47	1.20–1.79	<0.001
Missing	142	2.24	10	2.40			
Age at menarche (years)							
<12	1,103	17.38	63	15.14	1	(Referent)	
12	1,355	21.36	108	25.96	1.40	1.02–1.92	0.039
13	1,682	26.51	114	27.40	1.19	0.87–1.61	0.276
14	1,125	17.73	66	15.87	1.09	0.77–1.55	0.625
15+	1,026	16.17	63	15.14	1.17	0.83–1.66	0.365
Missing	54	0.85	2	0.48			
Race/ethnicity						29.15	
White	4,847	76.39	342	82.21	1	(Referent)	
Black	405	6.38	21	5.05	0.67	0.40–1.11	0.119
Hispanic	907	14.29	45	10.82	0.65	0.45–0.96	0.029
Asian	128	2.02	7	1.68	0.73	0.35–1.55	0.418
Missing	58	0.91	1	0.24			
Education, highest completed							
High school or general education development	3,077	48.49	164	39.42	1	(Referent)	
Vocational, technical, or some college or university	2,024	31.90	146	35.10	1.21	0.96–1.52	0.108
Bachelor or graduate degree	1,213	19.12	105	25.24	1.51	1.16–1.97	0.002
Missing	31	0.49	1	0.24			

Note: HRs are unadjusted but stratified by birth cohort (10-year groups) and study site. To account for clustering by family, robust 95% CIs are reported.

Abbreviations: *P*, nominal statistical significance; Q1–Q4, Quartiles 1–4.

### Statistical analysis

We fitted Cox proportional hazard models using age as the time axis to estimate the hazard ratios (HR) for the associations of weight, height, and BMI with breast cancer risk, stratified by study site and birth cohort, with considering underlying familial risk. On the basis of their distributions being skewed, and on maximizing the log likelihoods of fitted models following the principles of Box and Cox transformations (10), we log-transformed weight, BMI, and 1-year BOADICEA risk, and kept height on its natural scale, which were the scales of these variables used in the following analyses. In the base model, we adjusted for baseline age, history of benign breast disease, race/ethnicity, education, age at menarche, and 1-year BOADICEA risk as potential confounding factors due to the statistical significance of the associations with breast cancer risk being *P* < 0.05 when they were fitted by themselves. We calculated robust 95% confidence intervals (CI) that accounted for clustering by family and corresponding SE on the log(HR) scale. We used the log(HR)/SE to compare the strengths of association with each anthropometric measure in terms of differentiating cases from controls. All statistical tests were two-sided and estimates with a *P* value < 0.05 were considered nominally significant. We used Stata version 16 for all analyses (11).

We fitted models for each of weight, BMI, and height alone, and then separate models for each pairwise combination of weight, height, and BMI. We also adjusted for and fitted interactions with 1-year BOADICEA risk, i.e., the interactions between 1-year BOADICEA risk and each of BMI, weight, and height, and the interactions between 1-year BOADICEA risk and baseline age. In addition, BMI, weight, and height, 1-year BOADICEA risk and baseline age were all fitted as restricted cubic spline terms in the aforementioned Cox regression models to allow for the possible nonlinear relationships between these anthropometric measures and breast cancer risk (12).

The Akaike information criterion (AIC) was used to compare model fits (13); lower AIC indicates better fitting model. The difference in AIC, ΔAIC = AIC_i_ − AIC_min_, where AIC_i_ and AIC_min_ are the AIC values for a fitted model and a better model, respectively, indicates the information loss when a fitted model is used rather than the better model. The strength of evidence against the better-fitted model is considered to be substantial if 0 ≤ ΔAIC ≤ 2, considerably less if 4 ≤ ΔAIC ≤ 7, essentially none if ΔAIC > 10 (14). The likelihood ratio criterion (15) was used to test the hypothesis that the difference in the log likelihoods between two nested models is consistent with chance. The degrees of freedom in *χ*^2^ tests were one for anthropometric measures fitted as linear terms, and two for the measures fitted as restricted cubic spline terms. The log risk gradient for a variable in terms of change per SD was (2ΔLL)^0.5^, where ΔLL is the change in log likelihood from adding that parameter.

The following sensitivity analyses were conducted. First, we included only breast cancers known to be invasive. Second, we excluded *BRCA1* and *BRCA2* mutation carriers. Third, we excluded ever/current users of hormone replacement therapy. Fourth, we included BMI, weight, and 1-year BOADICEA risk on a natural scale.

### Ethics approval and consent to participate

All participants in the BCFR and kConFab provided written informed consent before cohort enrollment. Human research ethics committees at the participating institutions granted ethics approval for the six sites of the BCFR and for kConFab:

- Northern California – Cancer Prevention Institute of California, Institutional Review Board (2001–033) and Stanford University (45842), which ensure that all studies are conducted in accordance with the ethical principles of the Belmont Report.
- New York – Columbia University Medical Center, Institutional Review Board (AAA7794), which ensures that all studies are conducted in accordance with the ethical principles of US Common Rule and Declaration of Helsinki.
- Philadelphia – Fox Chase Cancer Center, Institutional Review Board (95–009), which ensures that all studies are conducted in accordance with the ethical principles of the Belmont Report.
- Utah – Huntsman Cancer Institute, University of Utah, Institutional Review Board (00004965), which ensures that all studies are conducted in accordance with the ethical principles of the Belmont Report and US Common Rule.
- Ontario – Mount Sinai Hospital Research Ethics Board (#02–0076-U) and University Health Network Research Ethics Board (#96-U107-CE), which ensure that all studies are conducted in accordance with the ethical principles of Declaration of Helsinki.
- Australia – University of Melbourne, Human Ethics Sub-Committee (1441420.1), which ensures that all studies are conducted in accordance with the ethical principles of Declaration of Helsinki.
- kConFab – Peter MacCallum Cancer Centre, the Peter Mac Ethics Committee (97/27), which ensures that all studies are conducted in accordance with the ethical principles of Declaration of Helsinki.

### Data availability

For access to the data used in this this study, please see http://www.bcfamilyregistry.org/ and www.kconfab.org.

## Results

Table 2 shows that, compared with the base model, the decreases in AIC were 16.40, 10.18, 3.93, when additionally including weight, BMI, and height, respectively. When included pairs of body measures BMI and height, weight and BMI, and weight and height, the decreases in AIC were 15.65, 15.50, and 15.57, respectively. Therefore, except for height alone, there was no evidence that the base model provided better fits than these measures. The AIC of the weight-only model was 6.22 smaller than the AIC of the BMI-only model, and provided considerably less support that BMI alone provided a better fit than the model using weight alone. The log risk gradient for weight was 1.23 times that for BMI. The ΔAICs between the weight-only model and the models using pairwise combinations of weight with BMI or height were less than two, indicating substantial evidence that these models all gave fits to a similar extent. However, the weight-only model included the least parameters, so weight alone gave the most parsimonious model fit.

**Table 2.** HRs and 95% CI for weight, BMI, and height with adjustment for 1-year BOADICEA risk.

Model^a^		HR^b^ 95% CI	*P*	ΔLL^c^	AIC
Weight	Log weight, kg	2.97 (1.87–4.71)	<0.001	9.20	4013.38
Height	Height, per 5 cm	1.10 (1.02–1.18)	0.017	2.96	4025.85
BMI	Log BMI, kg/m^2^	2.55 (1.54–4.22)	<0.001	6.09	4019.60
Weight and BMI	Log weight, kg	5.42 (1.51–19.51)	0.010	9.75	4014.28
	Log BMI, kg/m^2^	0.50 (0.13–1.99)	0.325		
Weight and height	Log weight, kg	2.70 (1.64–4.45)	<0.001	9.78	4014.21
	Height, per 5 cm	1.04 (0.96–1.13)	0.298		
Height and BMI	Height, per 5 cm	1.11 (1.03–1.20)	0.007	9.83	4014.13
	Log BMI, kg/m^2^	2.71 (1.65–4.47)	<0.001		

Note: To account for clustering by family, robust 95% CIs are reported.

^a^The *P*_interaction_ values between log 1-year BOADICEA risk and age at baseline were all >0.9.

^b^Adjusted for history of benign breast disease, race/ethnicity, education, and age at menarche; stratified by year of birth (10-year groups) and study site.

^c^ΔLL = change in log likelihood (LL) from the base model that includes baseline age, benign breast disease, race/ethnicity, education, age at menarche, and log (1-year BOADICEA risk).

In support of this, adding height or BMI to the weight-only model did not improve fit (both *P* for likelihood ratio test = 0.3; ΔAIC = 0.90 and 0.83, respectively). Conversely, adding weight to both the BMI-only and the height-only models improved fits (*P* = 0.007 and 0.0002, ΔAIC = 5.32 and 11.64, respectively). Adding BMI gave a better fit to the height-only model (*P* = 0.0002, ΔAIC = 11.72), and adding height also gave a better fit to the BMI-only model (*P* = 0.006, ΔAIC = 5.47).

The HR for familial risk was 1.93 (95% CI, 1.63–2.30) when fitted in the base model. While the HRs varied from 1.94 to 1.96 when additionally fitted with one or more of weight, BMI, and height, virtually no changes in the effects of familial risk were found. As previously found for BMI (7), for both weight and height, there was no evidence for multiplicative interactions with underlying familial risk (all *P* > 0.2).

Similar results were found when we excluded adjustment for familial risk (see Supplementary Table S1). The associations with weight, BMI, and height were also little changed after adjusting for familial risk, consistent with the lack of multiplicative interactions above.

Using the restricted cubic spline regression gave similar results, i.e., weight alone gave the most parsimonious model fit; BMI and height added information to the height-only model and BMI-only model, respectively; no evidence of multiplicative interactions between 1-year BOADICEA risk and weight or height or BMI (Supplementary Table S2). There was no evidence for nonlinear breast cancer risk associations with log-transformed BMI or weight, or height on a natural scale (*P* for nonlinear >0.2) (Supplementary Fig. S1). There was also no evidence of nonlinearity with 1-year BOADICEA risk or baseline age (*P* for nonlinearity >0.06; Supplementary Table S2).

### Sensitivity analyses

After adjusting for 1-year BOADICEA risk, in the models excluding *BRCA1* and *BRCA2* mutation carriers or restricted to invasive breast cancer or restricted to never users of hormone replacement therapy, results were similar, that is, (i) weight alone provided the most parsimonious model fit, and BMI and height added additional information to the models by themselves; (ii) there was no evidence for multiplicative interactions between 1-year BOADICEA risk and weight or BMI or height; (iii) there was evidence that log-transformed BMI or weight, or height on a natural scale, had linear associations with breast cancer risk (Supplementary Table S3, Supplementary Fig. S2). The aforementioned results still held when all the variables were analyzed on a natural scale, although the evidence was slightly weaker (Supplementary Tables S4 and S5, Supplementary Fig. S3), except for the marginal evidence for the nonlinear association between weight and breast cancer risk after adjusting for familial risk (*P* for nonlinearity = 0.049).

## Discussion

We found evidence that weight alone gives more information on breast cancer risk prediction for postmenopausal women than does BMI alone. None of the pairwise combinations of BMI, weight, and height provided more information on breast cancer risk than the weight-only model. On the contrary, while height added information to the BMI-only model, this did not provide a better fitting model than weight alone. This suggests that the adjustment of weight for height inherent in BMI is not optimal for breast cancer risk prediction. Therefore, BMI alone does not appear to capture the relationship between body measures and breast cancer risk as well as weight alone. The evidence for this was consistent across all the sensitivity analyses. Fitting log(BMI) corresponds to a special case of a model of log(weight) and log(height) in which the log(height) coefficient is forced to be in the opposite direction of the log(weight) coefficient, and of twice the absolute magnitude (16). We found no evidence for this (Supplementary Table S6); height was associated with an increased risk on its own and when it was fitted with BMI, and this association is well established (17). In addition, adding log(height) or log(weight) to log(BMI) will eliminate the constraint of the forced relationship between log(weight) and log(height) contained in log(BMI).

Similar observations have been found for breast cancer and other human traits. For example, a large-scale postmenopausal breast cancer study using the UK Biobank (18) found that the risk ratio per SD increase in weight was 1.24 (95% CI, 1.18–1.30; *χ*_1_^2^ = 74), while for BMI it was 1.21 (95% CI, 1.15–1.27; *χ*_1_^2^ = 42). Therefore ΔAIC = 32, providing substantial evidence that BMI alone gave a worse fit than weight alone. BMI alone also did not adequately describe the relationship of body composition and body size to systolic blood pressure for which weight was a better predictor (16).

These findings challenge current beliefs about the association between anthropometric measures and breast cancer risk. First, the World Cancer Research Fund International Continuous Update Project considered BMI and weight change, but not weight, as risk factors for breast cancer (19), and interpreted the association with BMI solely as a reflection of adiposity simply because it is a surrogate for adipose tissue. Our results, however, suggest that the relationship between BMI and breast cancer could instead be due to a relationship between weight (due to whatever source of tissue) and breast cancer, which is downgraded by inappropriately adjusting for height when using BMI. Therefore, using weight instead of BMI could avoid some biased conclusions in terms of postmenopausal breast cancer risk.

Second, our findings suggest the potential importance of anthropometric measures other than total body fat (20), rather than focusing on BMI simply because it is more strongly correlated with total body fat (21). These could be measures of body size, such as height, waist circumference, hip circumference, and fat-free mass, or measures of regional adiposity such as breast adiposity and abdominal adiposity (6, 22).

Third, our findings clarify that the breast cancer risk associations with BMI and weight, whether log-transformed or not, and height on its natural scale, are across a continuum and at least approximately linear, rather than a step function and restricted to the extreme and arbitrarily assigned categories labelled with negative connotations (23). Similar results have been found by a meta-analysis (24). Postmenopausal women classified in the normal or ‘healthy’ range of BMI between 20 and 25 kg/m^2^are taken to be at baseline risk and therefore not regarded as being at increased breast cancer risk, even though each increase in weight, and therefore BMI, could be associated with an increase in postmenopausal breast cancer risk. That is, the risk associations with weight and BMI are increasing across their ranges, and not a step-function as is implicit by only considering categories. Thus, describing breast cancer risk in terms of weight as a continuum, rather than in terms of BMI categories, would more accurately inform, and perhaps better translate, public health and clinical decisions relevant to breast cancer, i.e., no “healthy” category of BMI should be defined—each increase matters for both BMI and weight, in terms of postmenopausal breast cancer risk.

BMI, weight, height and familial risk are all positively associated with postmenopausal breast cancer risk. The lack of evidence for multiplicative interactions between the familial risk and BMI or weight or height in this study implies that, on the absolute scale, the additive positive associations of BMI or weight or height and the underlying familial risk with breast cancer risk must increase with any of them (7). This makes breast cancer risk management related to anthropometric measures and familial risk more important when they are considered together. More attention to weight is warranted for women with greater familial risk, while even for women in the lower categories of familial risk, greater weight could be problematic.

There are two main limitations in this study. One is that we did not consider risk separately by breast cancer subtypes defined by hormone receptor status, because these data were not available for a large proportion of cases. Risk associations with different subtypes of breast cancer vary according to BMI categories, which could reflect age and different etiologies (25, 26). The second is the wide CIs of the associations between breast cancer risk and body size measures in the restricted cubic spline regression; larger sample size is necessary to confirm the findings.

In conclusion, there was evidence that weight is more informative than BMI for predicting postmenopausal breast cancer risk, and breast cancer control based on body composition might need to be broader and more nuanced than just being focused on ‘adiposity’ and ‘obesity’. The contribution of weight to breast cancer risk would be more important the greater a woman's familial risk of breast cancer.

## Supplementary Material

Supplementary Data

## References

[1] Lauby-Secretan B , Scoccianti C, Loomis D, Grosse Y, Bianchini F, Straif K, . Body fatness and cancer–viewpoint of the IARC Working Group. N Engl J Med 2016;375:794–8. 27557308 10.1056/NEJMsr1606602 PMC6754861

[2] van den Brandt PA , Spiegelman D, Yaun S-S, Adami H-O, Beeson L, Folsom AR, . Pooled analysis of prospective cohort studies on height, weight, and breast cancer risk. Am J Epidemiol 2000;152:514–27. 10997541 10.1093/aje/152.6.514

[3] Michels KB , Greenland S, Rosner BA. Does body mass index adequately capture the relation of body composition and body size to health outcomes? Am J Epidemiol 1997;147:6. 10.1093/oxfordjournals.aje.a009430 9457007

[4] Neuhouser ML , Aragaki AK, Prentice RL, Manson JE, Chlebowski R, Carty CL, . Overweight, obesity, and postmenopausal invasive breast cancer risk: a secondary analysis of the women's health initiative randomized clinical trials. JAMA Oncol 2015;1:611–21. 26182172 10.1001/jamaoncol.2015.1546 PMC5070941

[5] Gallagher D , Heymsfield SB, Heo M, Jebb SA, Murgatroyd PR, Sakamoto Y. Healthy percentage body fat ranges: an approach for developing guidelines based on body mass index. Am J Clin Nutr 2000;72:694–701. 10966886 10.1093/ajcn/72.3.694

[6] Macinnis RJ , English DR, Gertig DM, Hopper JL, Giles GG. Body size and composition and risk of postmenopausal breast cancer. Cancer Epidemiol Biomarkers Prev 2004;13:2117–25. 15598769

[7] Hopper JL , Dite GS, MacInnis RJ, Liao Y, Zeinomar N, Knight JA, . Age-specific breast cancer risk by body mass index and familial risk: Prospective Family Study Cohort (ProF-SC). Breast Cancer Res 2018;20:132. 30390716 10.1186/s13058-018-1056-1 PMC6215632

[8] Terry MB , Phillips KA, Daly MB, John EM, Andrulis IL, Buys SS, . Cohort profile: the breast cancer Prospective Family Study Cohort (ProF-SC). Int J Epidemiol 2016;45:683–92. 26174520 10.1093/ije/dyv118 PMC5005937

[9] Antoniou AC , Cunningham AP, Peto J, Evans DG, Lalloo F, Narod SA, . The BOADICEA model of genetic susceptibility to breast and ovarian cancers: updates and extensions. Br J Cancer 2008;98:1457–66. 18349832 10.1038/sj.bjc.6604305 PMC2361716

[10] Box GE , Cox DR. An analysis of transformations. J R Stat 1964;26:211–43.

[11] StataCorp. Stata Statistical Software. Release 16 [software]. 2019 June [cited 2021 May 5]. Available from: https://download.stata.com/download/.

[12] Desquilbet L , Mariotti F. Dose-response analyses using restricted cubic spline functions in public health research. Stat Med 2010;29:1037–57. 20087875 10.1002/sim.3841

[13] Akaike H . A new look at the statistical model identification. IEEE Trans Autom Control 1974;19:716–23.

[14] Burnham KP , Anderson DR. Model selection and multimodel inference: a practical information-theoretic approach. 2nd ed. New York, NY: Springer; 2002. p. 70–2.

[15] Stuart A , Arnold S, Ord JK, O'Hagan A, Forster J. Kendall's advanced theory of statistics. Vol 2: Inference and Relationship. New York, NY: Hafner Publishing Company; 1961. p. 224–45.

[16] Karin B . Michels SG, Rosner BA. Does BMI adequately captured the relation of body composition and body size to health outcomes. Am J Epidemiol 1997;147:167–72. 10.1093/oxfordjournals.aje.a009430 9457007

[17] Zhang B , Shu X-O, Delahanty RJ, Zeng C, Michailidou K, Bolla MK, . Height and breast cancer risk: evidence from prospective studies and Mendelian randomization. J Natl Cancer Inst 2015;107:djv219. 26296642 10.1093/jnci/djv219 PMC4643630

[18] Guo W , Key TJ, Reeves GK. Adiposity and breast cancer risk in postmenopausal women: results from the UK Biobank Prospective Cohort. Int J Cancer 2018;143:1037–46. 29569713 10.1002/ijc.31394 PMC6099222

[19] Bandera EV , Fay SH, Giovannucci E, Leitzmann MF, Marklew R, McTiernan A, . The use and interpretation of anthropometric measures in cancer epidemiology: a perspective from the world cancer research fund international continuous update project. Int J Cancer 2016;139:2391–7. 27352197 10.1002/ijc.30248

[20] Clinton SK , Giovannucci EL, Hursting SD. World Cancer Research Fund and American Institute for Cancer Research. Diet, nutrition, physical activity and cancer: a global perspective. J Nutr 2020;150:663–71. 31758189 10.1093/jn/nxz268 PMC7317613

[21] Revicki DA , Israel RG. Relationship between body mass indices and measures of body adiposity. Am J Public Health 1986;76:992–4. 3728773 10.2105/ajph.76.8.992 PMC1646642

[22] Savolainen-Peltonen H , Vihma V, Leidenius M, Wang F, Turpeinen U, Hamalainen E, . Breast adipose tissue estrogen metabolism in postmenopausal women with or without breast cancer. J Clin Endocrinol Metab 2014;99:E2661–7. 25215559 10.1210/jc.2014-2550

[23] World Health Organization. Obesity: preventing and managing the global epidemic. 2000;894:i–xii. 11234459

[24] Liu K , Zhang W, Dai Z, Wang M, Tian T, Liu X, . Association between body mass index and breast cancer risk: evidence based on a dose-response meta-analysis. Cancer Manag Res 2018;10:143–51. 29403312 10.2147/CMAR.S144619 PMC5783020

[25] Gershuni V , Li YR, Williams AD, So A, Steel L, Carrigan E, . Breast cancer subtype distribution is different in normal weight, overweight, and obese women. Breast Cancer Res Treat 2017;163:375–81. 28293912 10.1007/s10549-017-4192-x

[26] Rose DP , Vona-Davis L. Interaction between menopausal status and obesity in affecting breast cancer risk. Maturitas 2010;66:33–8. 20181446 10.1016/j.maturitas.2010.01.019

