# Supplementary material for: Weight is More Informative than Body Mass Index for Predicting Postmenopausal Breast Cancer Risk: Prospective Family Study Cohort (ProF-SC)
Source: Cancer Prev Res (Phila). 2022 Mar 4;15(3):185–91. doi: 10.1158/1940-6207.CAPR-21-0164 (PMC8977841; doi:10.1158/1940-6207.CAPR-21-0164)
Supplement: Supplementary Data [file capr-21-0164_supplementary_tables_s1-s6_and_supplementary_figures_s1-s3_supp3.pdf]

Table S1. Hazard ratios and 95% confidence intervals (CI) for weight, body mass index and height without adjustment for 1-year BOADICEA risk

Model		HR [a]	95%CI	*P*	ΔLL [b]	AIC
Weight	Log weight, kg	2.78	1.75,4.41	<0.001	8.14	4091.51
Height	Height, per 5 cm	1.09	1.01,1.17	0.032	2.43	4102.93
BMI	Log BMI, kg/m^2	2.44	1.47,4.04	0.001	5.53	4096.74
Weight and BMI	Log weight, kg	4.65	1.28,16.97	0.020	8.55	4092.71
	Log BMI, kg/m^2	0.55	0.14,2.23	0.404		
Weight and height	Log weight, kg	2.57	1.55,4.23	<0.001	8.58	4092.65
	Height, per 5 cm	1.04	0.96,1.13	0.374		
Height and BMI	Height, per 5 cm	1.10	1.02,1.19	0.015	8.62	4092.55
	Log BMI, kg/m^2	2.58	1.56,4.25	<0.001		

To account for clustering by family, robust 95% CIs are reported;
[a] Adjusted for history of benign breast disease, race/ethnicity, education, and age at menarche; stratified by year of birth (10-year groups) and study site;
[b] ΔLL = change in log likelihood (LL) from the base model that includes baseline age, benign breast disease, race/ethnicity, education, and age at menarche.

Table S2. Model fits of height, weight and body mass index as restricted cubic spline terms in Cox regression model

	BMI	Weight	Height	BMI and height	BMI and weight	Weight and height
AIC	4021.49	4013.89	4027.71	4017.80	4016.33	4016.86
ΔLL [a]	6.14	9.94	3.03	9.98	10.72	10.46
P [b]	0.996	0.869	0.674			
P [c]	0.096	0.087	0.067	0.083	0.079	0.081
P [d]	0.782	0.771	0.845	0.801	0.787	0.783

[a] ΔLL = change in log likelihood (LL) from the base model that includes baseline age, history of benign breast disease, race/ethnicity, education, age at menarche and 1-year BOADICEA risk.
[b] The *P* values for the interaction between weight/height/BMI and 1-year BOADICEA risk, respectively;
[c] The *P* values for non-linearity of the association between 1-year BOADICEA risk and breast cancer risk, respectively;
[d] The *P* values for non-linearity of the association between baseline age and breast cancer risk, respectively.

Table S3. The Cox regression models with the 1-year BOADICEA risk including invasive breast cancer only, excluding *BRCA1*/*BRCA 2* mutation carriers, and HRT never-users only

Restrictions (N/n)	Model [a]	HR [b]	95%CI	P value	ΔLL [c]	AIC
Never-users of HRT (3115/165)	Log BMI, kg/m^2	2.41	1.07,5.42	0.034	2.13	1403.89
	Log weight, kg	3.02	1.47,6.20	0.003	3.84	1400.48
	Height, per 5 cm	1.12	1.01,1.25	0.028	1.94	1404.27
	Log weight, kg	8.17	1.49,44.91	0.016	4.46	1401.24
	Log BMI, kg/m^2	0.32	0.05,2.13	0.237		
	Height, per 5 cm	1.14	1.02,1.26	0.017	4.38	1401.38
	Log BMI, kg/m^2	2.58	1.15,5.77	0.021		
	Log weight, kg	2.59	1.16,5.81	0.021	4.42	1401.32
	Height, per 5 cm	1.07	0.95,1.20	0.253		
Non-*BRCA1*/ *BRCA 2* mutation carriers (6116/342)	Log BMI, kg/m^2	2.49	1.43,4.34	0.001	4.89	3575.71
	Log weight, kg	2.84	1.71,4.70	<0.001	7.13	3571.24
	Height, per 5 cm	1.09	1.00,1.18	0.054	2.02	3581.45
	Log weight, kg	4.64	1.11,19.40	0.036	7.44	3572.63
	Log BMI, kg/m^2	0.57	0.12,2.68	0.475		
	Height, per 5 cm	1.10	1.01,1.20	0.027	7.52	3572.45
	Log BMI, kg/m^2	2.64	1.52,4.58	0.001		
	Log weight, kg	2.63	1.51,4.56	0.001	7.47	3572.56
	Height, per 5 cm	1.04	0.95,1.14	0.439		
Invasive breast cancer only (6466/389)	Log BMI, kg/m^2	3.17	1.91,5.25	<0.001	8.83	3893.31
	Log weight, kg	3.71	2.04,6.77	<0.001	12.99	3887.01
	Height, per 5 cm	1.11	1.03,1.20	0.007	3.59	3903.81
	Log weight, kg	6.99	1.95,25.11	0.003	13.55	3885.88
	Log BMI, kg/m^2	0.49	0.13,1.89	0.302		
	Height, per 5 cm	1.13	1.05,1.22	0.002	13.61	3885.77

	Log BMI, kg/m^2	3.44	2.07,5.69	<0.001		
	Log weight, kg	3.43	2.07,5.68	<0.001	13.57	3885.84
	Height, per 5 cm	1.05	0.96,1.13	0.278		

N/n the number of total observations/the number of events;
To account for clustering by family, robust 95% CIs are reported;
[a] The *P* values for the interaction between weight/height/BMI and 1-year BOADICEA risk were all >0.2;
[b] Adjusted for history of benign breast disease, race/ethnicity, education, and age at menarche; stratified by year of birth (10-year groups) and study site;
[c] ΔLL = change in log likelihood (LL) from the base model that includes baseline age, history of benign breast disease, race/ethnicity, education, age at menarche and 1-year BOADICEA risk;
HRT Hormone replacement therapy.

Table S4. Hazard ratios and 95% confidence intervals (CI) for weight, body mass index and height on their natural scale without adjustment for 1-year BOADICEA risk

Model		HR[a]	95%CI	*P*	ΔLL[b]	AIC
Weight	Weight, per 5 kg	1.06	1.03,1.09	<0.001	7.14	4093.53
	Age at baseline, years	0.95	0.92,0.98	0.002		
Height	Height, per 5 cm	1.09	1.01,1.17	0.032	2.43	4102.93
	Age at baseline, years	0.95	0.93,0.98	0.003		
BMI	BMI, per 5 kg/m^2	1.16	1.07,1.26	<0.001	5.25	4097.31
	Age at baseline, years	0.95	0.92,0.98	0.001		
Weight and BMI	Weight, per 5 kg	1.09	1.00,1.19	0.049	7.32	4095.16
	BMI, per 5 kg/m^2	0.93	0.73,1.19	0.571		
	Age at baseline, years	0.95	0.92,0.98	0.002		
Weight and height	Weight, per 5 kg	1.06	1.03,1.09	<0.001	7.72	4094.35
	Height, per 5 cm	1.04	0.96,1.13	0.300		
	Age at baseline, years	0.95	0.93,0.98	0.002		
Height and BMI	Height, per 5 cm	1.10	1.02,1.19	0.016	8.27	4093.27
	BMI, per 5 kg/m^2	1.17	1.08,1.27	<0.001		
	Age at baseline, years	0.95	0.92,0.98	0.002		

To account for clustering by family, robust 95% CIs are reported;

[a] Adjusted for history of benign breast disease, race/ethnicity, education, and age at menarche; stratified by year of birth (10-year groups) and study site;

[b] ΔLL = change in log likelihood (LL) from the base model that includes baseline age, history of benign breast disease, race/ethnicity, education, and age at menarche.

Table S5. Hazard ratios and 95% confidence intervals (CI) for weight, body mass index and height on their natural scale with adjustment for 1-year BOADICEA risk

Model [a]		HR [b]	95%CI	*P*	ΔLL [c]	AIC
Weight	1-year BOADICEA (%)	3.86	1.87,7.97	<0.001	6.92	4016.94
	Age at baseline, years	0.96	0.93,0.99	0.011		
	Weight, per 5 kg	1.06	1.03,1.09	<0.001		
	1-year BOADICEA (%) × age at baseline, years	0.98	0.97,1.00	0.010		
Height	1-year BOADICEA (%)	4.32	2.10,8.87	<0.001	3.20	4024.38
	Age at baseline, years	0.96	0.93,0.99	0.019		
	Height, per 5 cm	1.10	1.02,1.19	0.013		
	1-year BOADICEA (%) × age at baseline, years	0.98	0.97,0.99	0.004		
BMI	1-year BOADICEA (%)	3.90	1.89,8.05	<0.001	4.73	4021.32
	Age at baseline, years	0.96	0.93,0.99	0.010		
	BMI, per 5 kg/m^2	1.15	1.06,1.25	0.001		
	1-year BOADICEA (%) × age at baseline, years	0.98	0.97,1.00	0.009		
Weight and BMI	1-year BOADICEA (%)	3.90	1.89,8.04	<0.001	7.30	4018.17
	Age at baseline, years	0.96	0.93,0.99	0.013		
	Weight, per 5 kg	1.10	1.01,1.20	0.027		
	BMI, per 5 kg/m^2	0.90	0.71,1.15	0.408		
	1-year BOADICEA (%) × age at baseline, years	0.98	0.97,1.00	0.009		
Weight and height	1-year BOADICEA (%)	3.93	1.90,8.12	<0.001	7.93	4016.92
	Age at baseline, years	0.96	0.93,0.99	0.014		
	Weight, per 5 kg	1.05	1.02,1.09	0.001		
	Height, per 5 cm	1.06	0.98,1.15	0.172		
	1-year BOADICEA (%) × age at baseline, years	0.98	0.97,1.00	0.009		
Height and BMI	1-year BOADICEA (%)	3.90	1.89,8.05	<0.001	8.51	4015.75
	Age at baseline, years	0.96	0.93,0.99	0.010		
	Height, per 5 cm	1.11	1.03,1.20	0.007		
	BMI, per 5 kg/m^2	1.16	1.07,1.26	<0.001		
	1-year BOADICEA (%) × age at baseline, years	0.98	0.97,1.00	0.009		

To account for clustering by family, robust 95% CIs are reported;

[a] The *P* values for the interaction between weight/height/BMI and 1-year BOADICEA risk were all >0.2;

[b] Adjusted for history of benign breast disease, race/ethnicity, education, and age at menarche; stratified by year of birth (10-year groups) and study site;

[c] ΔLL = change in log likelihood (LL) from the model that includes baseline age, history of benign breast disease, race/ethnicity, education, age at menarche and 1-year BOADICEA risk.

Table S6. Hazard ratios and 95% confidence intervals (CI) for log transformed weight, body mass index and height with adjustment for 1-year BOADICEA risk

Model [a]	HR [b]	95%CI	*P*	ΔLL [c]	AIC
Log BMI, kg/m^2	2.55	1.54, 4.22	<0.001	6.09	4019.60
Log weight, kg	2.97	1.87, 4.71	<0.001	9.20	4013.38
log height, cm	20.02	1.54, 260.71	0.022	2.90	4025.98
Log weight, kg	5.42	1.51, 19.51	0.010	9.75	4014.28
Log BMI, kg/m^2	0.50	0.13, 1.99	0.325		
log height, cm	29.37	2.27, 380.55	0.010	9.75	4014.28
Log BMI, kg/m^2	2.71	1.64, 4.47	<0.001		
Log weight, kg	2.71	1.64, 4.47	<0.001	9.75	4014.28
log height, cm	4.00	0.25, 63.03	0.325		

To account for clustering by family, robust 95% CIs are reported;

[a] The *P* values for the interaction between weight/height/BMI and 1-year BOADICEA risk were all >0.2;

[b] Adjusted for history of benign breast disease, race/ethnicity, education, and age at menarche; stratified by year of birth (10-year groups) and study site;

[c] ΔLL = change in log likelihood (LL) from the model that includes baseline age, history of benign breast disease, race/ethnicity, education, age at menarche and 1-year BOADICEA risk.

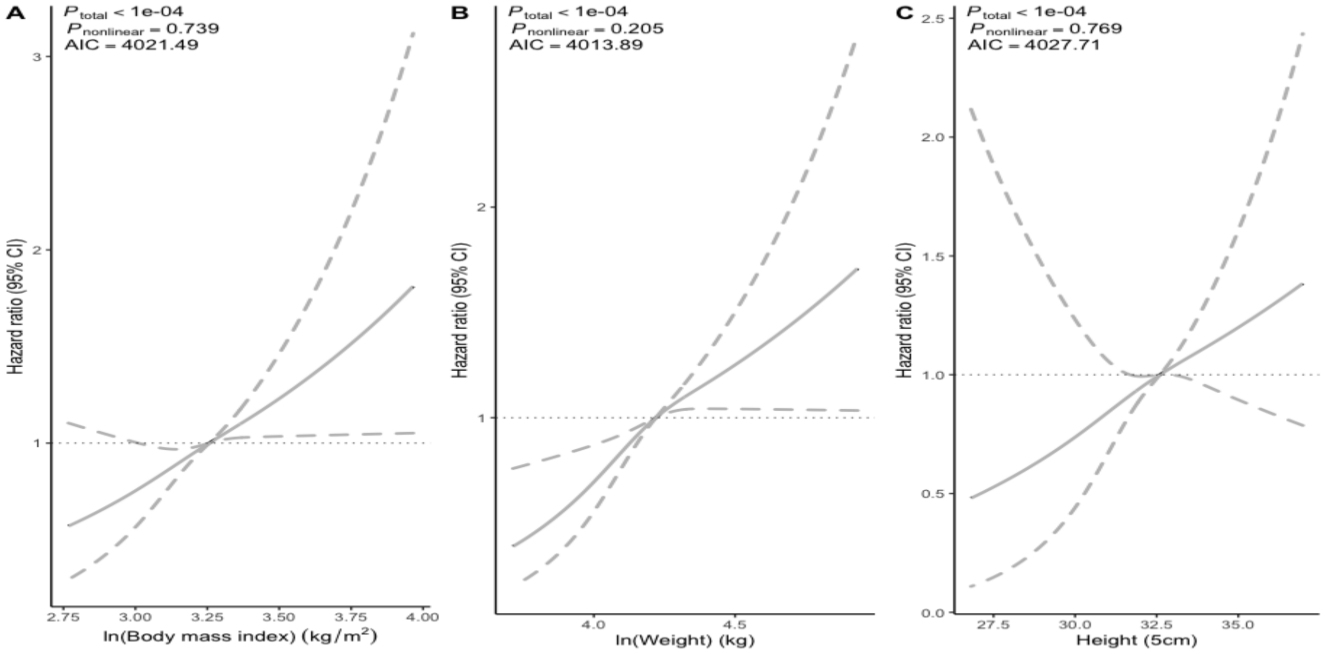

Figure S1. The association between BMI/weight/height and breast cancer risk with adjustment for familial risk

Footnote: BMI, weight and height were all fitted as restricted cubic spline terms in the Cox regression models to explore possible nonlinear relationships between these measures and breast cancer risk. Three knots were selected for each of the measures, i.e., 20, 25, 30 kg/m^2 for BMI; 52, 66, 79 kg for weight; and 150, 160, 170 cm for height, which divided the association between anthropometric measures and breast cancer risk into four parts. 23 kg/m^2, 55 kg, and 163 cm were chosen as the referent for BMI, weight, and height, respectively.

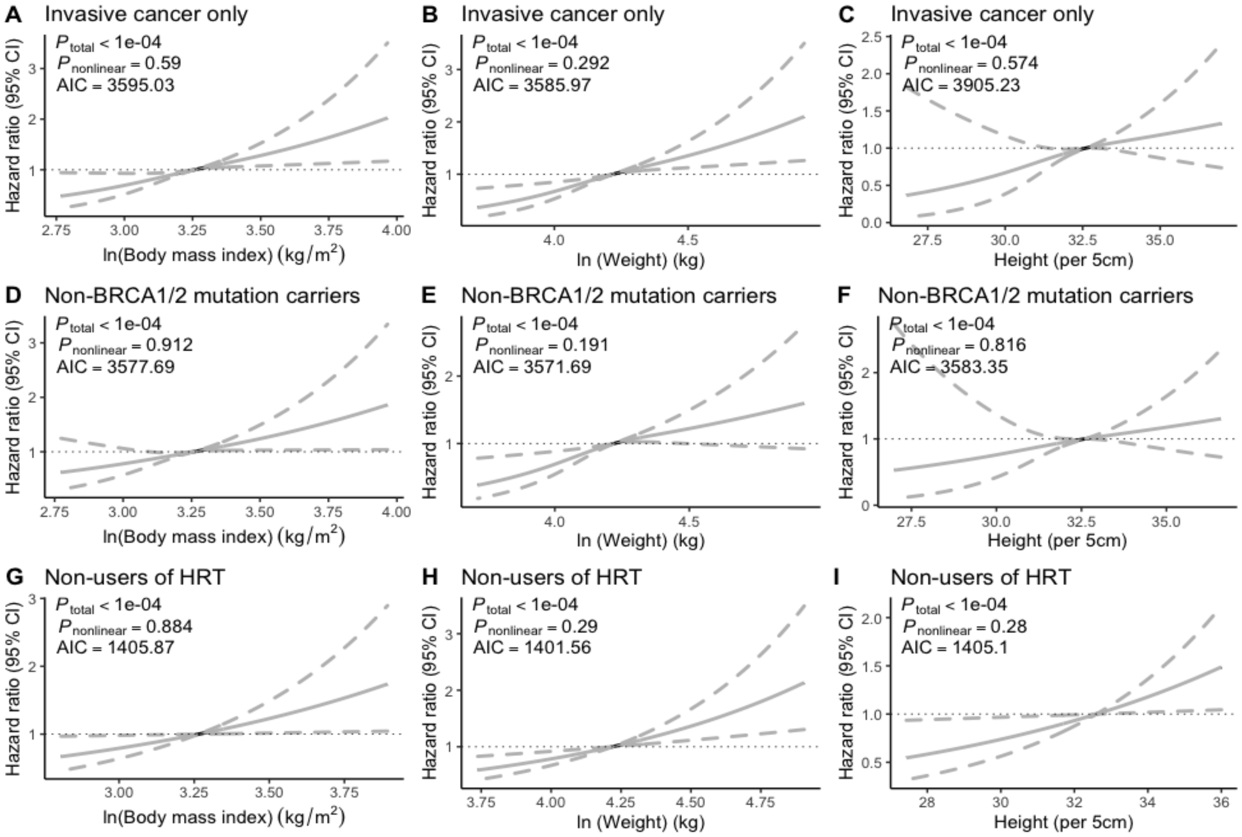

Figure S2. Association between body mass index/weight/height and breast cancer risk, adjusted for 1-year BOADICEA risk, restricted to invasive breast cancers only (Figure S2A-C, non-BRCA1/2 mutation carriers only (Figure S2D-F), and HRT never-users only (Figure S2G-I), respectively.

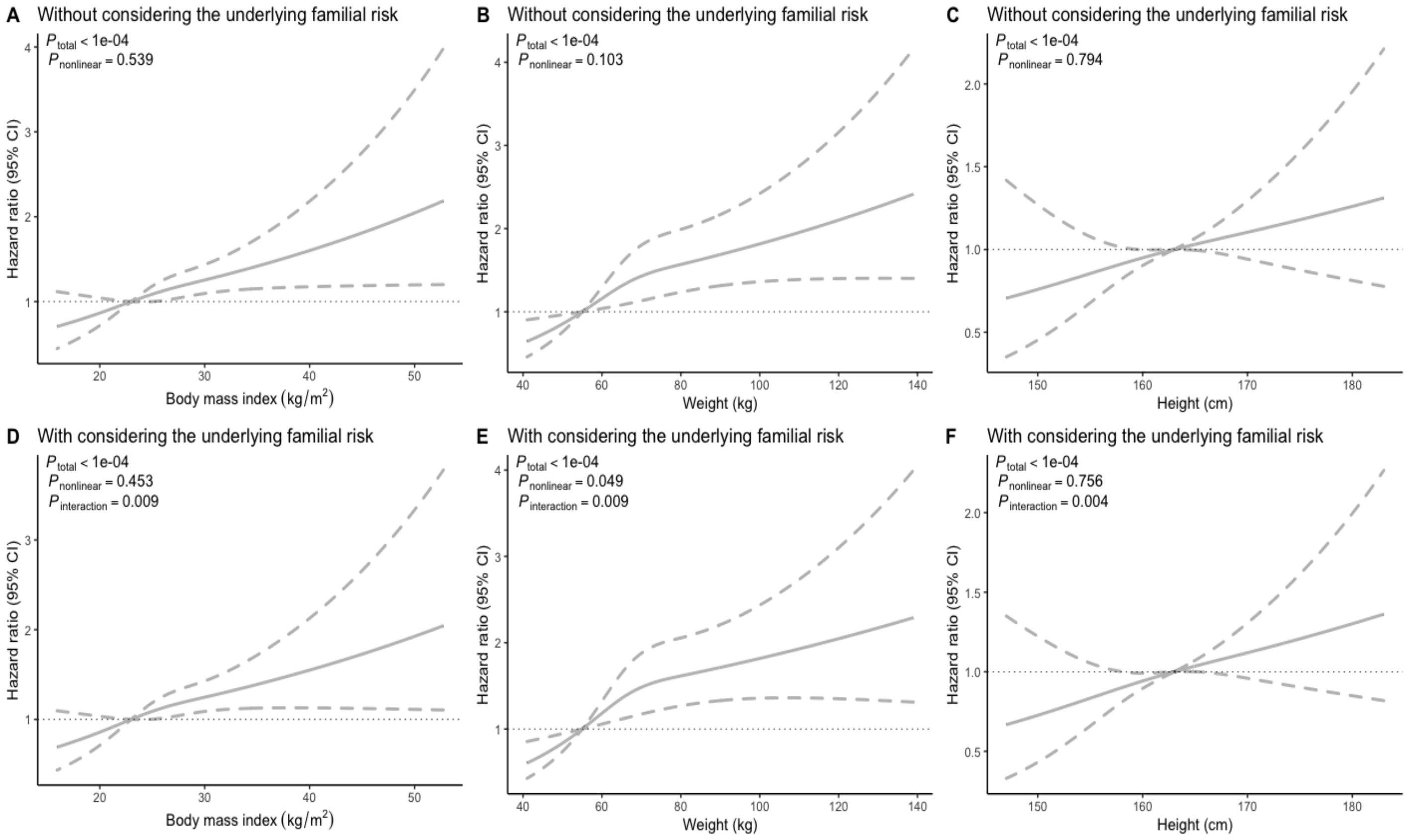

Figure S3. Association between body mass index/weight/height, on a natural scale, and breast cancer risk, with or without adjustment for 1-year BOADICEA risk
